# Drug survival of second biological DMARD therapy in patients with rheumatoid arthritis: a retrospective non-interventional cohort analysis

**DOI:** 10.1186/s12891-017-1684-0

**Published:** 2017-08-02

**Authors:** Thomas Wilke, Sabrina Mueller, Sze Chim Lee, Istvan Majer, Marieke Heisen

**Affiliations:** 1 IPAM, University of Wismar, Alter Holzhafen 19, 23966 Wismar, Germany; 2 Ingress-health, Alter Holzhafen 19, 23966 Wismar, Germany; 3 Pharmerit International, Marten Meesweg 107, 3068 Rotterdam, AV Netherlands

**Keywords:** Rheumatoid arthritis, bDMARD therapy, Anti-TNF, Continuation of bDMARD therapy, Switch of bDMARD therapy

## Abstract

**Background:**

Since persistence to first biological disease modifying anti-rheumatic drugs (bDMARDs) is far from ideal in rheumatoid arthritis (RA) patients, many do receive a second and/or third bDMARD treatment. However, little is known about treatment persistence of the second-line bDMARD and it is specifically unknown whether the mode of action of such a treatment is associated with different persistence rates. We aimed to assess discontinuation-, re-initiation- or continuation-rates of a 2nd bDMARD therapy as well as switching-rates to a third biological DMARD (3rd bDMARD) therapy in RA patients.

**Method:**

Analysis was based on German claims data (2010–2013). Patients were included if they had received at least one prescription for an anti-TNF and at least one follow-up prescription of a 2nd bDMARD different from the first anti-TNF. Patient follow-up started on the date of the first prescription for the 2nd bDMARD and lasted for 12 months or until a patient’s death.

**Results:**

2667 RA patients received at least one anti-TNF prescription. Of these, 451 patients received a second bDMARD (340 anti-TNF, mean age 52.6 years; 111 non-anti-TNF, mean age 55.9 years).

During the follow-up, 28.8% vs. 11.7% of the 2nd anti-TNF vs. non-anti-TNF patients (*p* < 0.001) switched to a 3rd bDMARD; 14.1% vs. 19.8% (*p* = 0.179) discontinued without re-start; 3.8% vs.1.8% (*p* = 0.387) re-started and 53.5 vs. 66.7% (*p* < 0.050) continued therapy. Patients in the non-anti-TNF group demonstrated longer drug survival (295 days) than patients in the anti-TNF group (264 days; *p* = 0.016).

Independent variables associated with earlier discontinuation (including re-start) or switch were prescription of an anti-TNF as 2nd bDMARD (HR = 1.512) and a higher comorbidity level (CCI, HR = 1.112), whereas previous painkiller medication (HR = 0.629) was associated with later discontinuation or switch.

**Conclusions:**

Only 56.8% of RA patients continued 2nd bDMARD treatment after 12 months; 60% if re-start was included. Non-anti-TNF patients had a higher probability of continuing 2nd bDMARD therapy.

**Electronic supplementary material:**

The online version of this article (doi:10.1186/s12891-017-1684-0) contains supplementary material, which is available to authorized users.

## Background

Rheumatoid arthritis (RA) is a chronic systemic autoimmune inflammatory disease with a prevalence of ≥1% in western countries [1, 2]. Poorly controlled RA results in severe progressive joint damage, functional disability, morbidity, poor health-related quality of life, and higher mortality than in the general population [3, 4].

Current treatment guidelines recommend methotrexate (MTX), a synthetic disease-modifying anti-rheumatic drug (sDMARD), in combination with glucocorticoids to be administered for most newly diagnosed RA patients [5, 6]. If MTX therapy fails to improve symptoms or fails to reach the treatment target, it is recommended to switch to another sDMARD (e.g., sulfasalazine, leflunomide, hydroxychloroquine) or to add a biological DMARD (bDMARD) to MTX therapy. Based on the mechanism of action, two broad classes of bDMARDs are distinguished: anti-TNFs (e.g., Infliximab, Etanercept, Adalimumab, Golumumab, Certolizumab) and non-anti TNFs (e.g., Abatacept, Anakinra, Tocilizumab, Rituximab) [7–9].

While most patients initially respond to first-line bDMARD, several studies reported that up to two thirds of the patients discontinue such a therapy after 12 months, mainly due to loss of efficacy and/or intolerable side effects [10–14]. A previous study showed that a substantial proportion of patients receives further lines of biologic treatment after such a discontinuation [15].

Since discontinuation and subsequent switching of treatments is mostly associated with either a treatment failure or serious adverse events, it has been frequently argued that long-term continuation of a particular bDMARD therapy (i.e., long drug survival) is a favourable outcome from both a clinical and economic perspective [10, 11, 16–22]. However, it is a matter of debate whether after a first anti-TNF therapy, treatment with a second-line anti-TNF or treatment with a non-anti-TNF biological is a strategy that maximizes drug survival of a second bDMARD therapy.

Previous studies assessing and comparing 2nd-line anti-TNF and non-anti TNF strategies in terms of drug survival are rare. So far, it has been found that less than 50% of the RA patients still continue a second-line bDMARD therapy after 1 year [14, 23–25]. Our study adds further evidence to this literature using a larger sample than most previous studies did.

In this analysis, we aimed to assess the drug survival of a second bDMARD therapy in RA patients hypothesizing that there is a difference in drug survival between a second anti-TNF versus non-anti-TNF therapy. By describing drug survival, we aimed to distinguish a switch to a 3rd-line bDMARD therapy from restart, discontinuation and continuation of a 2nd-line bDMARD therapy.

## Methods

A retrospective non-interventional cohort analysis was performed using anonymized medical insurance claims data provided by the AOK Plus, a German statutory health insurance fund with 3 million insured persons. German claims data have been found to be a reliable data source for health care analyses, especially those addressing outpatient drug treatment of patients [26–28].

Our analysis was done for the calendar years 2010–2013. RA patients were included if they were ≥18 years old, had ≥1 outpatient visit with an RA-related ICD-10 diagnosis code (M05 or M06, excluding M06.1: adult-onset still disease) and/or ≥1 hospital admission with an RA-related ICD-10 diagnosis code prior to the first prescription of an anti-TNF. Moreover, included patients should have had a prescription of a first anti-TNF treatment and, additionally, of a second bDMARD (either anti-TNF or non-anti TNF). Date of first prescription of the second bDMARD was defined as index date. Patients were selected for inclusion only if they were enrolled in the health insurance database at 01/01/2010 and could be followed for ≥12 months for drug survival after index date. Patients who died during this 12-month follow-up were included, however patients who received a Rituximab treatment as 2nd-line bDMARD treatment were described but excluded from the analysis because per label Rituximab treatment should be discontinued or delayed until disease activity returns, and information about disease activity was not available in our database [29].

In agreement with the study objective, the date of the first prescription of the second bDMARD was defined as the index date. For the purpose of the analysis, it was assumed that a patient discontinued a second bDMARD therapy if there was no documented prescription for ≥90 days (sensitivity analysis: ≥ 180 days) after the estimated end date of the drug supply. Based on the prescribed package size the duration of drug supply was estimated in terms of days according to the defined daily dose (DDD) as determined by the World Health Organization for each bDMARD (Additional file 1). If after an observed treatment gap of ≥90 days a patient received a new prescription of the same second bDMARD the patient was classified as a re-starting patient. Alternatively, a patient who received a new prescription for a third bDMARD was labeled as a switching patient. All remaining patients were classified 2nd-line bDMARD treatment continuers.

### Statistical analysis

Patient characteristics were obtained for and compared between the anti-TNF and non-anti-TNF groups. Characteristics measured as continuous variables were compared applying Mann-Whitney U test whereas categorical variables were compared using Fisher’s exact test. Patient populations were considered statistically different along a particular characteristic if the *P* value was lower than 0.05.

Total discontinuation rates were reported for the 12-month follow-up period for the anti-TNF and non-anti-TNF groups, and were reported separately for patients who restarted the second bDMARD therapy, who switched to a third bDMARD therapy, and who discontinued the second bDMARD without receiving any documented further biologic treatment. Drug survival of the second bDMARD treatment was estimated using the Kaplan-Meier method and compared between patients who received an anti-TNF versus a non-anti-TNF second bDMARD by means of log-rank tests. Both switching and discontinuation of 2nd-line bDMARD therapy were considered as an event indicating no drug survival. As restarting of a therapy follows on discontinuation of the same therapy, this was not considered a separate event on top of discontinuation.

To account for differences in patient characteristics between RA patients who received anti-TNFs versus non-anti TNFs as 2nd-line bDMARD, we estimated the hazard ratio (HR) of treatment discontinuation (non-anti-TNF versus anti-TNF) by multivariable Cox proportional hazards models. Again, both switching and discontinuation of 2nd-line bDMARD therapy was considered as an event. The following risk factors were initially included in the model and covariates were selected via backward elimination (*p* > 0.100): age, gender, Charlson Comorbidity Index (CCI) without age as factor, prescribed medications in the 6 months before 2nd-line bDMARD initiation (12 different ATC groups), concomitant sDMARD therapy, concomitant and previous painkiller prescriptions (anesthetics, analgesics, non-steroidal anti-inflammatory and antirheumatic agents and celecoxib with ATC codes: N01*, N02*, M01A*, L01XX33), concomitant corticosteroid prescriptions (glucocorticoids for systemic use and glucocorticoids combinations with ATC codes: H02AB*, H02BX*), and adverse events during first bDMARD therapy (see Additional file 2 for further details). Factors reaching a *p* < 0.05 were interpreted as statistically significant. All reported *p*-values were two-sided, and 95% CIs were calculated for Hazard Ratios (HRs). Descriptive evaluations were performed with Microsoft SQL Server 2013 and Microsoft Excel 2013. All other statistical analyses were performed with SPSS 17.0/STATA 13.1.

## Results

### Patient sample

We identified 2667 RA patients with a first observable anti-TNF prescription during 01/01/2010–31/12/2012 (Additional file 3). Among these, 495 patients received at least one prescription for a 2nd bDMARD (mean age: 53.9 years, 76.8% female, CCI 2.12, exposure time: 180,419 days; 364.5 days per patient); 340 had received an anti-TNF (116 Adalimumab, 42 Certolizumab, 120 Etanercept, 46 Golimumab, 16 Infliximab; mean age: 52.9 years, 77.4% female, CCI 2.11, exposure time: 123,844 days; 364.2 days per patient), and 155 had received a non-anti-TNF (40 Abatacept, 3 Anakinra, 68 Tocilizumab, 44 Rituximab, mean age: 56.2 years, 75.5% female, CCI 2.14, exposure time: 56,575 days; 365.0 days per patient). Because Rituximab patients were excluded from the study sample, the final dataset consisted of 451 RA patients (111 non-anti-TNF). The patients who received a 2nd anti-TNF were younger than the patients who received a 2nd non-anti-TNF (*p* = 0.055). Furthermore, patients who received a non-anti TNF also received corticosteroids both before and after index date with a higher probability. No significant differences between the 2nd anti-TNF and non-anti TNF groups could be observed for gender, CCI, duration of first-line anti-TNF treatment, and other pre-/post-index medication like sDMARDs, or painkillers/NSARs (Table 1).

**Table 1:** Characteristics of observed RA patient samples

Characteristics	Total patient population		Patients with anti-TNF as second bDMARD		Patients with non-anti-TNF as second bDMARD		*P* value (anti-TNF versus non-anti-TNF)
N	451	(100.00%)	340	(100.00%)	111	(100.00%)	
Age [in years], mean (SD)	53.75	(12.77)	52.96	(SD: 13.25)	56.17	(SD: 10.85)	0.055
Female gender, n (%)	351	(77.83%)	263	77.35%	88	79.28%	0.671
Mean CCI	2.09	(SD: 1.68)	2.11	(SD: 1.70)	2.03	(SD: 1.59)	0.786
First anti-TNF agent, n (%)							
• Adalimumab • Certolizumab • Etanercept • Golimumab • Infliximab	177 17 182 42 33	(39.25%) (3.77%) (40.35%) (9.31%) (7.32%)	136 10 136 30 28	(40.00%) (2.94%) (40.00%) (8.82%) (8.24%)	41 7 46 12 5	(36.94%) (6.31%) (41.44%) (10.81%) (4.50%)	0.566 0.106 0.788 0.532 0.190
Duration of first anti-TNF treatment [in days]							
• Mean (SD) • Median (range)	357.71 272.00	(SD: 265,16) (1061.00)	348.92 263.50	(SD: 263,81) (1039.00)	384.60 315.00	(SD: 268,67) (1059.00)	0.167
At least one prescription of … within 6 months before index date, n (%)							
• sDMARDs	216	(47.89%)	160	(47.06%)	56	(50.45%)	0.535
• Painkillers/NSARs	317	(70.29%)	234	(68.82%)	83	(74.77%)	0.234
• Corticosteroids	326	(72.28%)	230	(67.65%)	96	(86.49%)	<0.001
At least one prescription of … within 6 months after index date**, n (%)							
• sDMARDs	202	(44.79%)	157	(46.18%)	45	(40.54%)	0.300
• Painkillers/NSARs	313	(69.40%)	234	(68.82%)	79	(71.17%)	0.641
• Corticosteroids	318	(70.51%)	225	(66.18%)	93	(83.78%)	<0.001

Legend: This table depicts the characteristics of the patients who were observed during this study

Notes: Age is calculated at start of first anti-TNF treatment, CCI was calculated based on in- and outpatient diagnosis within 6 months before first anti-TNF agent was prescribed; in case first anti-TNF prescription was documented in the first half of 2010, the first 6 months of 2010 were used for CCI calculation

Abbreviations: *SD* standard deviation, *CCI* Charlson Comorbidity Index

### Assessment of 2nd bDMARD drug survival

Table 2 presents the proportion of patients who switched, discontinued (with and without later re-start) or remained on second bDMARD therapy during the 12-month follow-up period. In the full population, the switch, discontinuation, and continuation rates were estimated to be 24.6% (95% CI: 20.8–28.8), 18.8% (95% CI: 15.5–22.7), and 56.8% (95% CI: 52.1–61.3), respectively. Treatment continuation rates were significantly lower in the anti-TNF group (53.5%, 95% CI: 48.2–58.8) than in the non-anti-TNF group (66.7%, 95% CI: 57.3–74.9). This was mainly explained by the switch rates, which were significantly higher in the anti-TNF group than in the non-anti-TNF group, 28.8% (95% CI: 24.2–33.9) versus 11.7% (95% CI: 6.9–19.2) (*p* < 0.001), respectively. The 12-month discontinuation rate was somewhat lower in the anti-TNF group (17.9%, 95% CI: 14.2–22.4) than in the non-anti-TNF group (21.6%, 95% CI: 14.9–30.3), however, the difference was not statistically significant (*p* = 0.403).

**Table 2:** Switch, discontinuation, and continuation rates of second bDMARD therapies

	Full patient population [n (%, 95%-CI)]		Patients with anti-TNFs as second bDMARD [n (%, 95%-CI)]		Patients with non-anti-TNFs as second bDMARD [n (%, 95%-CI)]		*Difference in percentage points, p-value (anti-TNF* versus *non-anti-TNF)*
*Observed patients*	*451*	*(100.0%)*	*340*	*(100.0%)*	*111*	*(100.0%)*	
Switchers	111	(24.6%, 95%-CI: 20.8–28.8)	98	(28.8%, 95%-CI: 24.2–33.9)	13	(11.7%, 95%-CI: 6.9–19.2)	*−17.1%,* *p < 0.001*
Discontinuers (90 day gap)	85	(18.8%, 95%-CI: 15.5–22.7)	61	(17.9%, 95%-CI: 14.2–22.4)	24	(21.6%, 95%-CI: 14.9–30.3)	*3.7%,* *p = 0.403*
*Among discontinuers (90 day gap): patients who re-started therapy*	*15*	*(17.6%, 95%-CI: 10.8–27.5)*	*13*	*(21.3%, 95%-CI: 12.6–33.6)*	*2*	*(8.3%, 95%-CI: 2.0–29.0)*	*−13.0%,* *p = 0.158*
Continuers (90 day gap)	256	(56.8%, 95%-CI: 52.1–61.3)	182	(53.5%, 95%-CI: 48.2–58.8)	74	(66.7%, 95%-CI: 57.3–74.9)	*13.2%,* *p = 0.015*
Discontinuers (180 day gap)	67	(14.9%, 95%-CI: 11.9–18.5)	45	(13.2%, 95%-CI: 10.0–17.3)	22	(19.8%, 95%-CI: 13.4–28.3)	*6.6%,* *p = 0.093*
Continuers (180 day gap)	273	(60.5%, 95%-CI: 55.9–65.0)	197	(57.9%, 95%-CI: 52.6–63.1)	76	(68.5%, 95%-CI: 59.2–76.5)	*10.6%,* *p = 0.045*

Notes: Switcher: a patients who received a third bDMARD within 12 months after index date (in the anti-TNF group, prescribed 3rd bDMARD agents were Etanercept (23.5%), Tocilizumab (18.4%), Golimumab (17.3%), Adalimumab (15.3%), Abatacept (11.2%), Rituximab (7.1%), Certolizumab (5.1%), Anakinra (1.0%), and Infliximab (1.0%); in the non-anti-TNF group, prescribed 3rd bDMARD agents were Abatacept (38.5%), Tocilizumab (23.1%), Golimumab (15.4%), Etanercept (7.7%), Rituximab (7.7%), and Certolizumab (7.7%)); Discontinuer: a patient who discontinued the second bDMARD with or without re-starting the treatment after a 90 days / 180 days of treatment gap, Re-starter: a patient who received at least one prescription of the second bDMARD agent (same agent) after a treatment discontinuation; Continuer: a patient who neither switched nor discontinued the second bDMARD treatment during the 12 months follow up

Note that one patient belonging to the anti-TNF group switched his second bDMARD therapy after a previous discontinuation and re-start. Here, we opted for double-counting, so that this patient belongs to both the re-starter and the switch group

In the sensitivity analysis applying a discontinuation threshold of ≥180 days, 12-month continuation rates for the overall sample were higher than in the main analysis (60.5% instead of 56.7%). Nevertheless, the proportion of patients who continued therapy was still higher in the non-anti TNF group (68.5%, 95% CI: 59.2–76.5) compared to the anti-TNF group (57.9%, 95% CI: 52.6–63.1).

**Fig. 1:**
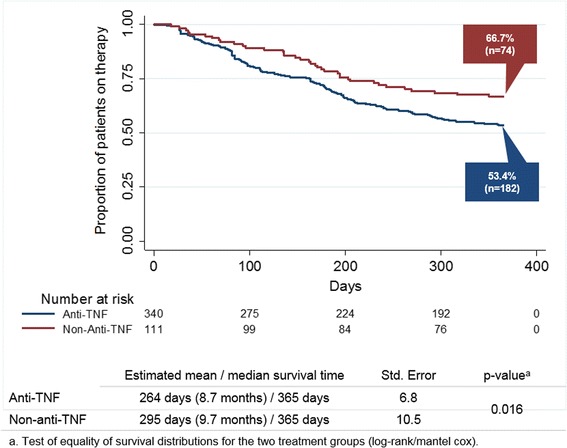
12-month drug survival of 2nd bDMARD therapy in RA patients. Shows 12-month drug survival of RA patients having initiated a 2nd bDMARD therapy. Patients died during the observation period were censored. Differences between patients having received an anti-TNF versus those having received a non-anti-TNF were analysed using Log-Rank tests

Figure 1 depicts second bDMARD drug survival rates for the anti-TNF and non-anti-TNF populations for the first year after initiation of a second bDMARD therapy as KM curves. Patients belonging to the non-anti TNF group had a greater chance to continue their second bDMARD therapy than patients belonging to the anti-TNF group. This holds true for the entire follow-up period; LogRank test (*p* = 0.016) showed this difference was statistical significant. Estimated mean drug survival time for the first year after initiation of a second bDMARD was 294.7 days (95% CI: 274.1–315.3 days) in the anti-TNF group and 263.8 days (95% CI: 250.6–277.2 days) in the non-anti-TNF group.

### Factors associated with 2nd bDMARD drug survival

**Fig. 2:**
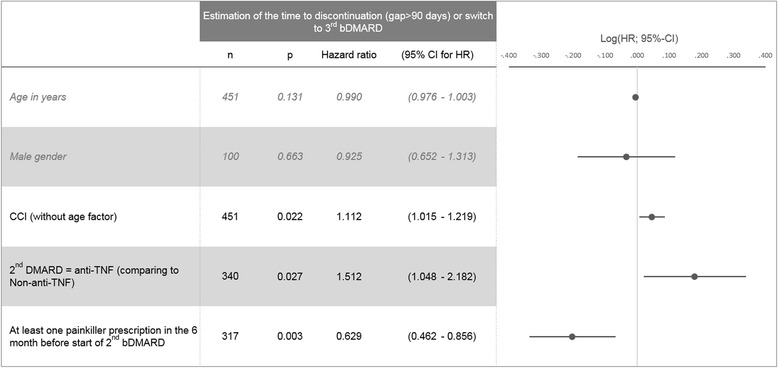
Predictors of 2nd bDMARD drug discontinuation. Estimation was done using multivariable Cox regression analysis based on a backward inclusion methodology (all variables with *p* < 0.100 were included). The model was controlled for age, gender and previous prescribed medications (differentiated by 12 different ATC main groups). Excluded variables were: concomitant DMARD therapy, concomitant painkiller medication, concomitant corticosteroid medication, adverse events before index date as defined in Additional file 2. Results are shown as 95%-CIs

In the multivariable Cox regression analysis, several patient and treatment characteristics were associated with the time to switch or discontinuation of the 2nd bDMARD therapy (Fig. 2). Patients who were more comorbid as measured by the CCI had a higher risk of discontinuing the 2nd bDMARD therapy or switching to a 3rd bDMARD therapy (HR = 1.112; 95% CI: 1.015–1.219, HR associated with CCI score points). In contrast, patients who had received at least one prescription for a painkiller in the 6 preceding months before index date had a lower risk of discontinuing or switching therapy (HR = 0.629; 95% CI: 0.462–0.856). Age and gender were not significantly associated with the probability of switching or discontinuing 2nd bDMARD treatment. Finally, patients who started a 2nd bDMARD therapy with an anti-TNF had a higher risk of a switch or discontinuation of therapy than patients who started with a non-anti-TNF (HR = 1.512; 95% CI: 1.048–2.182).

## Discussion

### Study objectives and main results

Using a German claims dataset, we aimed to assess the level of drug survival in RA patients who started a 2nd bDMARD therapy after discontinuation of a 1st anti-TNF therapy, and to compare drug survival between patients who received an anti-TNF and those who received a non-anti-TNF as a 2nd bDMARD.

We reported that a substantial number of RA patients discontinued or switched their 2nd bDMARD therapy within 12 months. Overall, only 56.8% of the patients continued their 2nd bDMARD therapy during a 12-month follow-up. Among those not continuing the 2nd bDMARD therapy, 56.6% switched to a 3rd bDMARD therapy, 35.7% discontinued therapy, and the remaining 7.7% re-started therapy after discontinuation. This percentage of re-starters may be related to the relatively short follow-up period of our study.

This study is one of the first studies analyzing drug survival of a 2nd bDMARD therapy in RA patients. Moreover, it is the first analysis covering Germany in this respect. A specific characteristic of our study is that it reported the percentage of patients who switched, discontinued (with and without re-start), or continued therapy separately. This allows a better understanding and interpretation of drug survival observed in clinical practice settings. Moreover, because we performed analyses using claims data instead of prospective or registry data, the selection bias of our study can be considered to be minimal.

We acknowledge that discontinuation of a bDMARD therapy or switching to another bDMARD agent may not directly imply therapy failure or serious adverse events. Due to the nature of the dataset, determination of disease activity and identification of any causes of adverse events was not possible, and thus we were not able to determine the specific reasons for therapy discontinuation or switches of therapy. However, it is known from existing literature that approximately 50% of observed bDMARD therapy switches/discontinuations are due to lack of disease remission under this therapy, whereas the remaining 50% are due to serious side effects [18, 20, 30, 31]. Thus, we assumed that our observed discontinuation and switch rates are a reliable proxy to describe the effectiveness and safety of different 2nd bDMARD therapies in RA.

### Comparison of results with existing publications

Our reported 12-month 2nd bDMARD discontinuation rates of between 53.5% (anti-TNF) and 66.7% (non-anti-TNF) are in line with existing literature. However, the majority of past studies were based on US data and almost exclusively covered 1st bDMARD therapy in RA patients. These studies reported 1st bDMARD 12-month therapy continuation rates of 62.2–68.9% [12], 42–56% [10, 11], 24–59% [13], and 48–51% [14], and the differences in the reported rates could be mainly due to different study designs, definitions of therapy discontinuation and patient sample characteristics. In Europe, an Italian analysis reported a likelihood of continuing 1st anti-TNF therapy of 78.8% after 12 months, 65.2% after 24 months, and 52.9% after 36 months, with a risk of dropout similar for inefficacy and adverse events as discontinuation causes [21]. A Greek analysis reported 5-year 1st bDMARD continuation rates of 31–49% [32]. In our study, we initially included all RA patients with at least one anti-TNF prescription to further identify patients with a second bDMARD. Of all 2667 anti-TNF patients, only 495 patients (18.5%) received a second bDMARD. This might indicate a good efficacy/safety of a first anti-TNF therapy. However, in line with our methodology, we did not define any index date or minimum follow-up period for the first anti-TNF therapy. Moreover, patients who discontinued a first anti-TNF therapy but did not start a second bDMARD therapy were not specifically identified. So, based on our numbers, no reliable assessment of first anti-TNF persistence is possible.

Much less is known about drug survival of 2nd bDMARD treatment in RA patients. A Spanish analysis reported DAS-28-based EULAR [33] response levels to 1st anti-TNF of 42% (good) compared to 20% for 2nd anti-TNF therapy, which could indicate a lower continuation rate of a 2nd anti-TNF therapy compared to a 1st anti-TNF therapy [23]. Another US publication reported 30–36 weeks 2nd bDMARD continuation rates of 46–56% [14].

In our study, neither sex nor age was associated with 2nd bDMARD drug survival, which was also shown in a Canadian analysis [24], whereas a Spanish single-centre analysis found age to be associated with therapy continuation [25]. In our analysis, previous painkiller medication was associated with a lower probability of discontinuing 2nd bDMARD treatment. To our knowledge, no such relationship has been found in previous studies. If painkiller medications are associated with severity of RA symptoms, this could mean that patients experiencing more or more severe RA symptoms have a higher probability of continuing their 2nd bDMARD treatment.

Additionally, in our analysis a higher comorbidity status and a 2nd anti-TNF treatment compared to a non-anti-TNF therapy were associated with an earlier 2nd bDMARD switch or discontinuation. This was also shown by most of the previous studies [10, 19, 30].

### Limitations

We acknowledge several limitations of our analyses. Due to the nature of our dataset, we could only observe a 12 months follow-up period after the start of 2nd bDMARD therapy. We also assumed that the first observed anti-TNF therapy in a patient was equivalent to the first-line bDMARD therapy this patient received. It is unknown whether some of the patients had already received other bDMARDs in previous unobserved periods, which could mean that we interpreted a bDMARD therapy to be a 2nd bDMARD therapy also when it was a therapy beyond the second line.

We defined discontinuation as a treatment gap of at least 90 days. Because most of bDMARD package sizes cover a DDD-based drug supply of 30–90 days, we identified the first therapy discontinuers 120–180 days after therapy start. While the threshold of 90 days is widely used in the literature [13], it has so far hardly been clinically validated. We dealt with this weakness by additionally using a threshold of at least 180 days. Our results show, as expected, that reported discontinuation rates decreased on the basis of this threshold, albeit only by a small percentage (14.9% discontinuation rate based on 180-day gap versus 18.8% based on 90-day gap).

In our analysis, which addressed outpatient bDMARD drug treatment only, we assumed that the start date of a therapy was equal to the date of filling a bDMARD prescription. If the application of the drug was done some days later than that, we assumed an earlier therapy start date than actually implemented. Moreover, stop date of a therapy was calculated based on the provided drug supply of each prescription. If packages were not used completely, we nevertheless assumed that the therapy was continued until the provided package was completely used. In addition to that, primary non-adherence (patient received prescription, but did not fill it) was not included into our analysis because of the nature of the dataset.

Additionally, we had only limited data with regard to potential factors associated with 2nd bDMARD discontinuation. Detailed data about RA disease severity and disease characteristics which may predict treatment outcomes and, consequently, treatment continuation were not available. So, due to unobserved patient and treatment characteristics, the results of the comparison of drug survival between anti-TNF and non-anti-TNF agents may be biased.

## Conclusions

### Conclusions and implications for practice

Over 50% of German RA patients discontinue their 2nd bDMARD treatment within 12 months after initiation. Patients on non-anti TNF biological DMARDs seem to have a higher drug survival and seem less likely to switch their therapy. We presume that, after an anti-TNF failure, this finding is related to the different mode of action of non-anti TNFs.

### Key messages

At least 50% of RA patients discontinue the 2nd bDMARD treatment within 12 months after initiation.

Patients on non-anti TNF bDMARDs as 2nd bDMARD therapy have a lower probability to discontinue their treatment.

## Additional files

Additional file 1: DDD-based drug supply of different agents that were available in the German market 2010–2013. (DOCX 15 kb)

Additional file 2: Observed adverse effects before initiation of a second bDMARD treatment (used proxies). (DOCX 14 kb)

Additional file 3: Flow diagram. This figure depicts the flow of patients, who were included in this study. (TIFF 709 kb)

## Abbreviations

ATC: Anatomical therapeutic chemical
bDMARD: Biological disease-modifying anti-rheumatic drug
CCI: Charlson comorbidity index
DDD: Defined daily dosage
GER: German
HR: Hazard ratio
ICD: International statistical classification of diseases
KM: Kaplan Meier
MOA: Mode of action
MTX: Methotrexate
RA: Rheumatoid arthritis
sDMARD: Synthetic disease-modifying anti-rheumatic drug
TNF: Tumour necrosis factor-alpha
