# Supplementary material for: Drug survival of second biological DMARD therapy in patients with rheumatoid arthritis: a retrospective non-interventional cohort analysis
Source: BMC Musculoskelet Disord. 2017 Aug 2;18:332. doi: 10.1186/s12891-017-1684-0 (PMC5540414; doi:10.1186/s12891-017-1684-0)
Supplement: Supplementary file 1 — DDD-based drug supply of different agents that were available in the German market 2010–2013. (DOCX 15 kb) [file 12891_2017_1684_MOESM1_ESM.docx]

***Additional file 1: DDD-based drug supply of different agents that were available in the German market 2010-2013***

| **Agent class** | **Agent** | **ATC code** | **Days’ supply (based on DDD)** | | | **Dosage according to product label** |
| --- | --- | --- | --- | --- | --- | --- |
|  |  |  | **Pack size N1** | **Pack size N2** | **Pack size N3** |  |
| Anti-TNF | Adalimumab | L04AB04 | 27  *(injection solutions: 50 days)* | 55 | 82 | Bi-weekly |
|  | Certolizumab | L04AB05 | - | 28 | 85 | 200 mg: bi-weekly  400mg: monthly |
|  | Etanercept | L04AB01 | 14 | 28 | 85 | 25 mg: twice a week  50 mg: once a week |
|  | Golimumab | L04AB06 | 30 | 90 | - | Monthly |
|  | Infliximab | L04AB02 | 80 | 133 | - | Every 8 weeks |
| Non-anti TNF | Abatacept | L04AA24 | 18 | 27 | - | Monthly |
|  | Tocilizumab | L04AC07 | 80 mg: 4  200 mg: 10  400 mg: 20 | 80 mg: 16  200 mg: 40  400 mg: 80 | - | Weekly |
|  | *Rituximab^[[1]](#footnote-1)^* | *L01XC02* | *2 1000 mg intravenous infusions; need for further courses should be evaluated 24 weeks following the previous course - retreatment should be given at that time if residual disease activity remains, otherwise retreatment should be delayed until disease activity returns* | | | |

1. Patients having received Rituximab were not included in the analysis. [↑](#footnote-ref-1)
