# Supplementary material for: Drug survival of second biological DMARD therapy in patients with rheumatoid arthritis: a retrospective non-interventional cohort analysis
Source: BMC Musculoskelet Disord. 2017 Aug 2;18:332. doi: 10.1186/s12891-017-1684-0 (PMC5540414; doi:10.1186/s12891-017-1684-0)
Supplement: Supplementary file 2 — Observed adverse effects before initiation of a second bDMARD treatment (used proxies). (DOCX 14 kb) [file 12891_2017_1684_MOESM2_ESM.docx]

***Additional file 2: Observed adverse effects before initiation of a second bDMARD treatment (used proxies)***

| ***Used proxies for 2^nd^ bDMARD-related AEs: AEs assumed to have happened if therapy was switched to a 2nd agent and a new inpatient or outpatient diagnosis of … was documented in the quarter in which the second bDMARD was first prescribed or in the previous quarter*** |
| --- |
| … Infections of the upper respiratory tract (including tracheitis, nasopharyngitis, pharyngitis, laryngitis, rhinitis, sinusitis)  (ICD-10 code: J00.*-J06.*) … |
| … Infections of the lower respiratory tract (including bronchitis)  (ICD-10 code: J20.*-J22) … |
| … Pneumonia  (ICD-10 code: J10.0, J12.*-J18.*) … |
| … Sepsis (ICD-10 code: T80.2, A40.*, A41.*) … |
| … Influenza (ICD-10 code: J09.*, J10.*) … |
| … Candidiasis (ICD-10 code: B37.*) … |
| … Urinary tract infections (including Pyelonephritis) (ICD-10 code: N10, N39.0) … |
| … Herpes infections (including herpes simplex, oral herpes and herpes zoster)  (ICD-10 code: B00.*) … |
| … Bacterial infections  (ICD-10 code: A31.*, A36.*-AA39) … |
| … Listeriosis (inclunding Listeriensepsis)  (ICD-10 code: A32.*) … |
| … Legionaires´ disease  (ICD-10 code: A48.1/A48.2) … |
| … Skin and soft tissue infections (including paronychia, cellulitis, impetigo, necrotizing fasciitis) (ICD-10 code: L00.*-L08.*, M60.0, M63.*, M65.1, M68.0*, M71.1*, M72.6*) … |
| … Fungal infections (including Coccidioidomycosis, Hystoplasmosis, Blastomycosis, Aspergillosis) (ICD-10 code: B35.*-B49.*) … |
| … Tuberculosis (ICD-10 code: A15.*-A19.*) … |
| … Skin cancer except melanoma (including basal cell carcinoma and squamous) (ICD-10 code: C44.* (M805-M808; M809-M811)) … |
| … Lymphoma (ICD-10 code: C81.*-C86.*) … |
| … Lymphadenopathy (ICD-10 code: R59.*) … |
| … Neutropenia / agranulocytosis  (ICD-10 code: D70.*) … |
| … Anemia (ICD-10 code: D60.*-D62.*) … |
| … Leukocytosis (ICD-10 code: D72.8) … |
| … Thrombocytopenia  (ICD-10 code: D69.4*-D69.6*) … |
| … Paresthesia (ICD-10 code: R20.2) … |
| … Allergies / anaphylaxis  (ICD-10 code: T78.2, T78.4) … |
| … Conjunctivitis (ICD-10 code: H13.1*) … |
| … Blepharitis (ICD-10 code: H01.0) … |
| … Flush (ICD-10 code: R23.2) … |
| … Psychiatric disorders (including depression, anxiety, insomnia)  (ICD-10 code: F32.*, F41.*, F51.*) … |
| … Migraine (ICD-10 code: G43.*) … |
| … Tachycardia (ICD-10 code: I47.*) … |
| … Cough (ICD-10 code: R05) … |
| … Dyspnea (ICD-10 code: R06.0) … |
| … Dyspepsia (ICD-10 code: K30) … |
| … Gastroesophageal Reflux Disease  (ICD-10 code: K21.*) … |
| … Sicca syndrome (ICD-10 code: M35.0) … |
| … Stomatitis (ICD-10 code: K12.*) … |
| … Hepatitis (ICD-10 code: B15.*-B19.*) … |
| … Rash (including eczema, dermatitis, pruritus) (ICD-10 code: L20.*-L30.*) … |
