# Supplementary figures and images for: Drug survival of second biological DMARD therapy in patients with rheumatoid arthritis: a retrospective non-interventional cohort analysis
Source: BMC Musculoskelet Disord. 2017 Aug 2;18:332. doi: 10.1186/s12891-017-1684-0 (PMC5540414; doi:10.1186/s12891-017-1684-0)

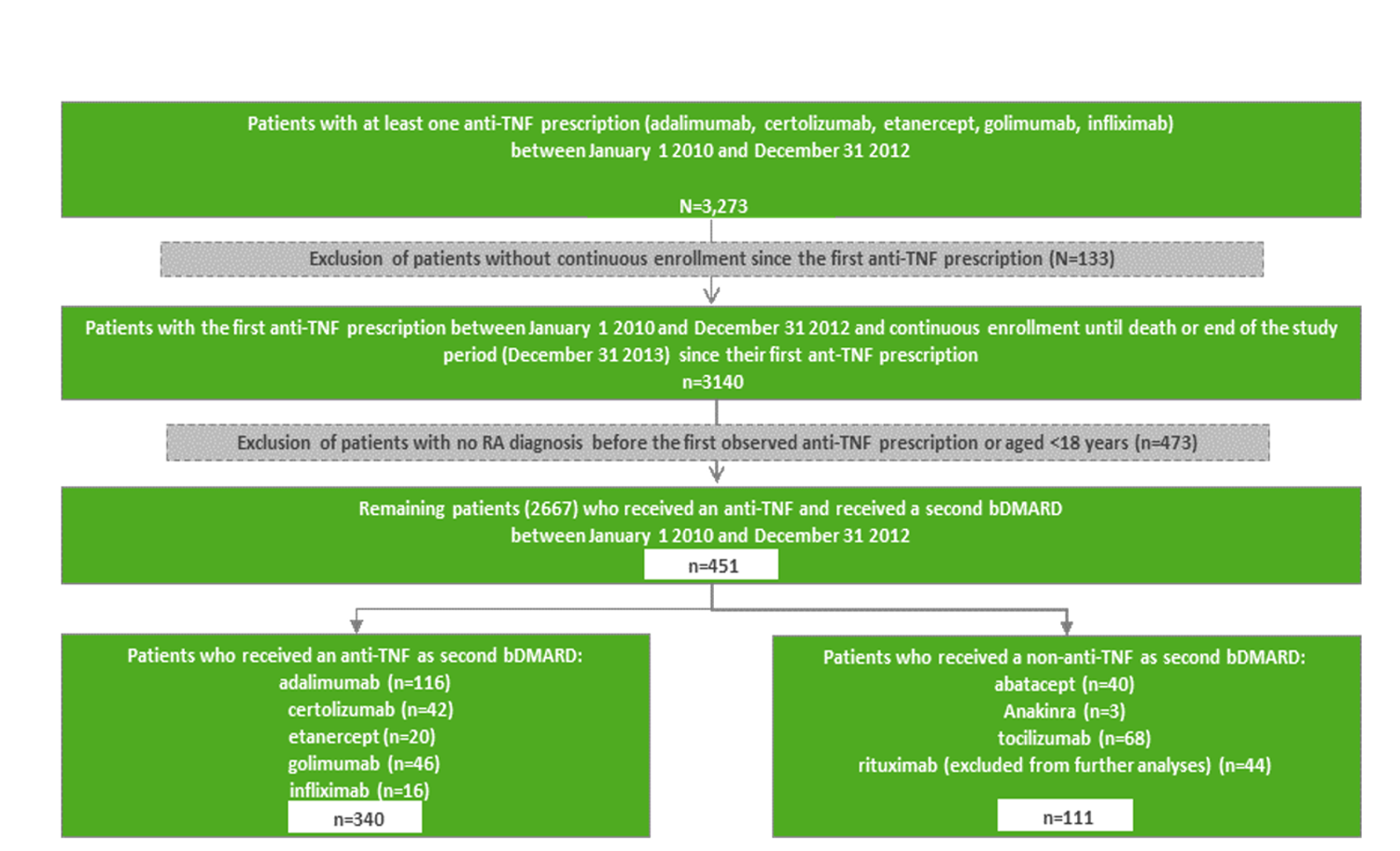

Supplement: Supplementary file 3 — Flow diagram. This figure depicts the flow of patients, who were included in this study. (TIFF 709 kb) [file 12891_2017_1684_MOESM3_ESM.tif]
